# The ecology of mosquitoes in an irrigated vegetable farm in Kumasi, Ghana: abundance, productivity and survivorship

**DOI:** 10.1186/1756-3305-5-233

**Published:** 2012-10-15

**Authors:** Yaw A Afrane, Bernard W Lawson, Ruth Brenya, Thomas Kruppa, Guiyun Yan

**Affiliations:** 1 School of Health Sciences, Bondo University College, Bondo, Kenya; 2 Kumasi Centre for Collaborative Research into Tropical Medicine, Kwame Nkrumah University of Science and Technology, Kumasi, Ghana; 3 Department of Theoretical and Applied Biology, Kwame Nkrumah University of Science and Technology, Kumasi, Ghana; 4 School of Medical Sciences, Kwame Nkrumah University of Science and Technology, Kumasi, Ghana; 5 Program in Public Health, College of Health Sciences, University of California, Irvine, CA, 92697, USA

**Keywords:** Urban malaria, Irrigated farms, *Anopheles gambiae*, Mosquito productivity, Kumasi, Ghana

## Abstract

**Background:**

Irrigated vegetable farms within the city of Kumasi, Ghana, create hotspots for the breeding of malaria vectors, which could lead to high transmission of malaria. This study investigated the abundance and productivity of mosquitoes in an irrigated vegetable farm in Kumasi, Ghana.

**Methods:**

Adult mosquito productivity was estimated five days in a week in different irrigated scheme types (dug-out wells, furrows and footprints) for 12 weeks using emergence traps. Larval sampling was done five days a week to estimate the abundance of larvae from the different irrigated schemes types.

**Results:**

Mosquito breeding in the irrigated vegetable field was confined to dug-out wells, furrows and human footprints. Mosquito productivity (m^2^/week) was highest in the dugout wells followed by the human footprints and the least was in the furrows (11.23, 5.07 and 4.34 *An. gambiae*/m^2^/week). Larval abundance for the late instars (3^rd^, 4^th^ and pupae) also followed the same trend, with the dug-out wells having the highest larval abundance followed by the human footprints and then the furrows (13.24, 6.81, 5.87 larvae/week). Mosquito productivity and abundance was negatively correlated with rainfall (R^2^ = 0.209; P< 0.01).

**Conclusion:**

This study showed that adult and larval mosquito abundance and larval survival were high in the irrigated fields in the irrigated vegetable farm. This therefore, contributed significantly to adult mosquito populations and hence malaria transmission in the city.

## Background

Malaria transmission levels in cities are normally less than those in the rural areas [1,2] because the highly polluted city waters do not allow for the breeding of malaria mosquitoes [2,3]. However, previous studies in the city of Kumasi showed that irrigated vegetable farms within the city are responsible for the production of over 80% of malaria mosquitoes in the city [4]. Consequently, the irrigated areas have mosquito numbers and malaria transmission levels that are similar to those of the surrounding rural areas. These vegetable farms are responsible for the production of 90% of vegetables consumed in the city [5]. In order to sustain the production of vegetables all year round, the farmers employ makeshift irrigation schemes. These farms are found in low-lying riverine basins where the water table is very high. The farmers dig shallow wells to reach the high water tables or construct conduits to divert water from nearby streams onto their farms for irrigation. The dug-out wells are also linked by furrows to make it easier for farmers to draw water with watering cans. The dug-out wells, furrows and human footprints in these irrigation schemes serve as breeding habitats for malaria vectors, making such irrigated areas have high numbers of adult malaria vectors and high malaria transmission levels [4].

Vector control is still the most practical method for reducing malaria transmission in developing countries [6,7]. A potentially important target of malaria vector control, especially in an urban setting, where breeding sites are few or confined to few places especially during the dry season, is the immature stages of the anopheline mosquitoes [7,8]. Control of aquatic-stage *Anopheles sp*. is one of the oldest and most historically successful interventions to prevent malaria, but it has seen little application in Africa [9].

Malaria transmission is dependent on the productivity of female *Anopheles* mosquitoes from suitable breeding habitats. The mosquito productivity in such habitats ultimately determines the density of anophelines. The actual number of adult vectors emerging from breeding habitats rather than high densities of larvae and pupae is considered medically important since only adult mosquitoes are able to disperse from the habitats and feed on human hosts [10]. Methods aimed at estimating the numbers of emerging adult mosquitoes thus are appropriate for determining productivity of mosquito breeding habitats. Larval survival and abundance as well as habitat productivity can be influenced by factors such as female oviposition preference [11] and habitat type, since the occurrence of predators is influenced by habitat size [12], type and conditions. Water must be available in a breeding site for at least ten days to support mosquito larvae in completing its aquatic life cycle [11,13].

In an urban environment *Anopheles* mosquitoes adapt to new breeding sites created by urbanization, and hence their ecology might differ from rural settings [14]. Most African studies *on Anopheles* mosquito larval ecology have been conducted in rural settings and findings from these studies might not be applicable to urban settings without adaptation [15]. However, a precise knowledge of the geographic location and potential of ecological characteristics of breeding sites is of major importance for such interventions.

The aim of the present study, therefore, was to investigate the abundance of immature mosquitoes, their survival and development as well as the production of adult mosquitoes in these irrigation schemes. The results of this study helped understand the mechanisms that regulate the populations of adult mosquitoes emerging from the vegetable farms and to exploit ways to undertake cost effective larval control in the irrigation farms in Kumasi.

## Methods

### Study site

The study site was an irrigated vegetable farm located at Gyinyase in the city of Kumasi, Ghana. This irrigation farm is about 37 hectares and is the largest of the irrigated vegetable farms in the city which all have similar construction. The farmers used ground water coming from bubbling springs in shallow wells for irrigating their farms. It is a low lying area close to the Wiwi River and the water table is therefore, very high. Mosquitoes breed in dug-out wells, furrows and human footprints which are created by the farmers as a result of their activities.

### Adult mosquito abundance in irrigated fields

Adult mosquito productivity (emerging adult mosquitoes) was estimated five days in a week in the different irrigated scheme types (dug-out wells, furrows and footprints) using emergence traps made from wood and covered with a nylon netting [16-18]. Five each of the dugout wells, furrows and human foot prints were sampled daily. Different dugout wells, furrows and footprints were sampled each week. The emergence traps had sleeves on two sides through which aspirators were inserted into the net to collect emerged adult mosquitoes. Traps measuring 1 x 1 m^2^, 1 x ½ m^2^, or ½ x ½ m^2^ were placed on top of the mosquito breeding habitats daily for the entire period of the study (February-May, 2006). Depending on the size of the habitat the appropriate trap size was used. Smaller traps were used for smaller habitats and the bigger traps were used for larger habitats. The emergence trap prevents adult mosquitoes from ovipositing in the area covered with the trap and immature mosquitoes from entering the trap; therefore, the emptied trap was daily relocated to different habitats. Although the traps may not provide an absolute estimation of mosquito productivity or abundance of a habitat, it is suitable for comparing the relative productivity of different larval habitat types [16,17,19]. Emerging adults were collected the following day with aspirators, counted and the number recorded. The mosquitoes were preserved and identified morphologically using the key of Gilles and De Meillon [20]. Adult mosquito abundance was calculated as number of emerging adults/m^2^/week [18].

### Mosquito larval abundance and predators in the irrigated fields

Mosquito breeding habitats comprising the dug-out wells, furrows and human foot prints were sampled daily for larval mosquito abundance. Dipping to sample larvae and pupae was done using the WHO 350 mL standard dipper. Larval and pupal numbers were recorded each time and specimens were brought to the laboratory for subsequent breeding to adult stage for morphological identification, using the key of Gilles and De Meillon [20]. This was done five times in a week.

The various breeding sites were described according to habitat characteristics such as degree of exposure to sunlight, and presence or absence of vegetation in the water, type of vegetation, occurrence of emergent plants, occurrence of algae, canopy cover, water depth etc. Five readings of depth and width of each habitat were taken and mean values calculated. An inventory of predators was made in each of the breeding sites sampled. Predators were identified according to literature [21-23]. There were no tests done to determine whether predators had actually fed on larvae or not. Daily rainfall data for the study area was obtained from the nearby meteorological station of the Kumasi Centre for Collaborative Research into Tropical Medicine (KCCR) in Kumasi, Ghana.

### Farmers’ use of pesticides, their knowledge about mosquitoes, malaria and larval control

Four focus group discussions involving four to six farmers each were conducted to obtain information on farmers’ use of insecticides and their frequency of use, farmers’ knowledge on mosquitoes and malaria as well as their willingness to cooperate and participate in larval control. The focus group discussion is a qualitative method for assessment of perceptions and general knowledge, in a format where a facilitator prompts participants to discuss the topic without answering a specific set of questions [24]. Farmers who have their plots near each other were asked to come together for this purpose. In-depth interviews were conducted among 18 out of 30 farmers to augment the focus group discussions. Overall, almost every farmer was involved in either the focus group discussion or in-depth interviews or both. Questions asked were written in English but were translated into Twi, the local language. All answers given in the focus group discussion as well as the in-depth interviews were transcribed in booklets and later typed onto a computer.

These studies were undertaken during the dry and rainy season of 2007 (February – May).

### Data analysis

Differences between mosquito abundance and productivity between different habitat type was analysed using Chi-square test. Correlation analysis was done to determine the relationship between various larval habitat characteristics and the occurrence of anopheline mosquito larvae. The presence or absence of mosquito larvae was used instead of absolute numbers of the larvae. Presence of larvae was taken as one and absence was zero. The analysis was conducted using JMP statistical software [25].

## Results

### Productivity of adult *An. gambiae*

**Figure 1:**
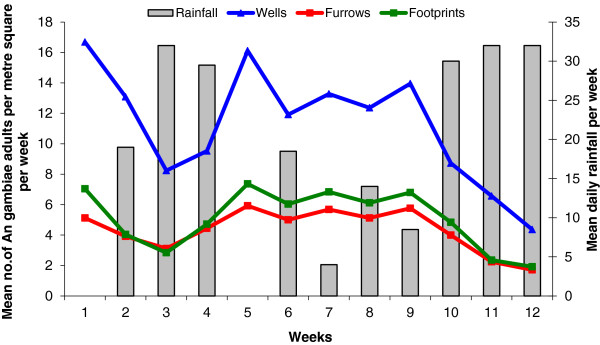
**Productivity of adult *An. gambiae *in three different breeding habitats in vegetable farms in Gyinyase, Kumasi, Ghana.**

A total of 6160 mosquitoes were collected with the emergence traps during the study period. Out of the total number, 4186 (67.9%) were *A. gambiae* and 1974 (32.1%) were culicines (belonging to the subfamily Culicinae). Culicines were not identified to species level for all individuals. Mosquito productivity per metre square per week was highest in the dugout wells followed by the human foot prints and was lowest in the furrows (11.23, 5.07 and 4.34 *An. gambiae*/metre square/week respectively; χ^2^=18.49, df=2, P< 0.0001; Table 1). Figure 1 illustrates the productivity of *An. gambiae* in the dug-out wells, furrows and human footprints during the study period. Water in the furrows was flowing most of the time whilst water in the footprints and dug-out wells was stagnant. There was, in general, a pattern of decrease in mosquito productivity with rainfall. Mosquito productivity was negatively correlated with rainfall (R^2^ = 0.209; P< 0.01).

**Table 1:** **Abundance of *An. gambiae *adult and immature forms in the irrigated field in Gyinyase**

	**Mean adult mosquito nos. m**^**2**^**/week ± S.D**	**Mean early instars mosquito nos. m**^**2**^**/week ± S.D**	**Mean late instars mosquito nos. m**^**2**^**/week ± S.D**
Dug-out wells	11.23 ± 3.78 ^a^	16.19 ± 3.87 ^a^	13.24 ± 3.86 ^a^
Foot prints	5.07 ± 1.93 ^b^	9.55 ± 2.41 ^b^	6.81 ± 2.21 ^b^
Furrows	4.34 ± 1.38 ^c^	8.96 ± 2.19 ^c^	5.87 ± 1.74 ^c^

^a, b,c^ -Different letters denote significant difference at P < 0.0001.

Note. – Standard deviation for adult abundance, early instars and late instar mosquitoes are shown.

### Abundance of immature mosquitoes

**Figure 2:**
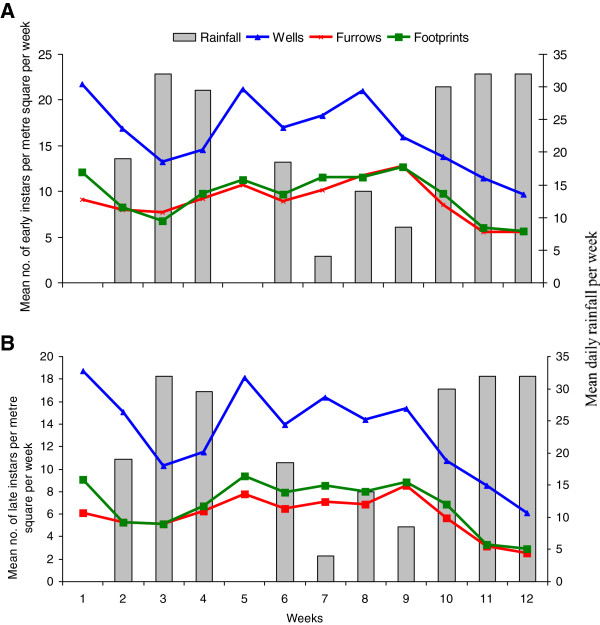
**Abundance of immature forms of *An. gambiae *in different breeding habitats: (A) Early instars (1st and 2nd instars); (B) Late instars (3rd and 4th instars) and pupae.**

A total of 5238 immature anophelines and 2390 culicines were encountered in this study. Abundance of the early larval instars (1^st^ and 2^nd^ instars) was highest in the dug-out wells followed by the footprints and then the furrows (16.19, 9.55 and 8.96 larvae/week; Table 1; χ^2^=18.57, df=2, P< 0.0001). Abundance of the late larval instars (3^rd^, 4^th^ and pupae) also followed the same trend, with the dug-out wells having the highest larval abundance followed by the human footprints and then the furrows (13.24, 6.81, 5.87 larvae/week; χ =18.29, df=2 P< 0.0001; Table 1). These results are also shown in Figure 2a and 2b. The anopheline larvae were allowed to grow to become adults in the insectary and then identified as *An. gambiae* s.l.

Rainfall was found to be negatively associated with both early and late instar larval abundance with the effect being higher in the early instars. The presence of algae and vegetation in the water significantly correlated positively with the presence of larvae. Habitats with debris of leaves had very little or no larvae. These results are illustrated in Table 2. At any fixed point in time the population of larvae in the breeding sites were composed of individuals of mixed age groups. There was also continual recruitment into the habitats because of the continuous laying of eggs by adult mosquitoes.

**Table 2:** **Correlation between the occurrence of *Anopheles gambiae *larvae and biotic and abiotic factors in the mosquito habitats**

**Instar**	**Rainfall**	**Algae**	**Vegetation in water**	**Debris cover**
**Early instar**	−0.978	0.609 ^ns^	0.517 ^ns^	−0.009 ^ns^
**Late instar**	0.462**	0.618 ^ns^	0.416 ^ns^	0.001 ^ns^

** Significant at the P<0.01 level.

^ns^ No significant difference.

### Predators in the breeding sites

Predators found in the breeding habitats included Amphibians (frogs and the immature forms the tadpoles) and arthropods, mainly insects (especially ants) and spiders. Frogs and tadpoles were mainly found in the dug-out wells but not in the furrows and footprints. Aquatic predators found included dragonfly nymphs and beetle larvae. These were found in the furrows and dug-out wells but not in the footprints. These insects have all been implicated as predators in larval breeding habitats [18,21,22]. Spiders were frequently encountered in the mud and vegetation at the edges of the larval habitats and on the water surface. Service [21] observed spiders preying on adults of *An. gambiae* in rice fields in western Kenya as the latter emerged from their pupal cases. Ants which have also been documented as potential predators [21] were found around the habitats. Their presence on all sampling visits suggests that they could be preying on stranded larvae from the breeding habitats.

### Farmers’ use of pesticides, their knowledge about mosquitoes, malaria and larval control

All farmers who participated in the in-depth interviews and focus group discussions were men. They cultivated lettuce, carrots, sweet pepper, onions and cabbage. The farmers use diathane for spraying their vegetables against fungal attack. They apply the fungicide every five days. Herbicides such as kalach and weedout were also used against weeds.

It was evident from the focus group discussions that the farmers did not know that their water sources bred mosquitoes, although 67% (12 out of 18) knew that mosquitoes 'cause' malaria. At least two people in each focus group discussion and 12 out of 18 farmers interviewed said that they knew that mosquitoes “cause” malaria. Participants noted that their water use and farming practices were necessary for economic survival and not designed to create larval habitats. None of the farmers had seen a mosquito larva before. However, when shown one, two respondents in one focus group said they sometimes see “some of these tiny insects” in the water. The farmers also admitted lack of knowledge on how mosquitoes could be controlled in their farm water sources. However, they expressed their willingness to the use of larvicides on condition that these would cause no harm to them or their vegetables. All the farmers said they would be willing to apply these larvicides on their farm if supplied to them.

## Discussion

This study showed that adult and larval mosquito abundance was high in the irrigated fields in the Gyinyase irrigated vegetable farm. Dug-out wells which served as the reservoirs for irrigation water had the highest larval abundance and mosquito productivity, followed by the footprints and then the furrows. Human footprints were seen to be present for a long time and were able to support larval breeding. Most mosquito larval breeding habitats in Africa are a result of anthropogenic environmental studies such as those described here.

Conditions in these breeding habitats are suitable for the survival and development of immature mosquitoes. The study site was sunlit and the irrigation water in the dug-out wells and furrows were clean, conditions which support the breeding of *An. gambiae.* Most habitats also had algal cover with less grass cover. These explain the high larval abundance, which also translated to a high production of adult mosquitoes. *An. gambiae* also prefers small, open habitats for oviposition rather than large habitats [12,21]. *An. gambiae* complex normally exploits the increased resources of warmer open habitats that tend to produce more algae (the main food source for the *An. gambiae* complex) than do shaded habitats [26]. Small habitats such as the dug out wells, furrows and the human footprints tend to have warmer temperatures and this shortens larvae-to-pupae development time while also reducing mortality associated with desiccation [20]. The *An. gambiae* complex may have evolved to exploit these favorable conditions by selecting small and open habitats for oviposition. It has been demonstrated in lowland areas of western Kenya, that habitat size is an important determinant of habitat stability, pupal occurrence, and mosquito abundance [27].

Just like studies by Munga *et al.,*[18] in western Kenya, mosquito productivity was high in the irrigation water sources at Gyinyase. It was further shown that habitat type affected productivity of adult *An. gambiae* s.l. It was observed that water in the habitats normally does not dry up because water seeps continuously from the soil below. Thus the immature mosquitoes are able to develop to become adults. The vegetable farmers employ poultry manure on their farms. The manure gets washed into the mosquito breeding waters and contributes to the high algal growth in these habitats. Few predators were found in the habitats suggesting that larval death due to predation might be minimal. These factors also explain the high productivity of mosquitoes in the irrigation scheme.

Rainfall was found to correlate negatively with both adult and larval abundance. The breeding habitats rely on water seeping from the ground and thus, rainfall, especially when heavy, rather washes the larvae and the pupae away leading to low mosquito productivity and larval abundance. Munga *et al.,*[18] also reported that higher amounts of rainfall washed larvae out of habitats, and thus reduced the abundance of mosquito larvae in western Kenya. According to these authors, this led to a reduced number of positive larval habitats during the rainy season.

The results of the present study have implications for the control of larval mosquitoes in the city of Kumasi. Knowledge of *An. gambiae* larval habitat productivity is important in planning and designing mosquito larval control interventions in such irrigated areas [9]. Since it was observed from the present study that mosquito breeding sites were confined to only the dug-out wells, furrows and human footprints, it makes it easier to apply larval control measures to these breeding sites. This will help reduce the number of adult mosquitoes in the area and thus potentially reduce malaria transmission. However, in most times resources for vector control are limited and thus it is always useful to look for a target to implement control measures [28]. Since it was found that dug-out wells are the most productive of all habitats, this could be targeted for vector control. When larvicides or bacteria formulations are applied to the dug-out wells their effects could be felt in the other irrigation water reservoirs such as the furrows and human foot prints. Besides, since it is water from the dug-out wells that also fills the furrows and the human footprints, targeting the dugout wells will be the best option in a resource limited situation.

The focus group discussions brought to the fore some important issues. The major challenges to community involvement in mosquito larval source reduction activities are in educating people about the sources of the mosquitoes and motivating people to assume responsibility for controlling mosquitoes in and around their homes [29,30]. These responsibilities are often assumed to be that of the government. In Dakar, Senegal, farmers who operate market garden wells in the city were required by law to have the fish *Gambusia sp*., which is a mosquito larval predator, in their wells [31]. This fish is supposed to prey on *An. arabiensis* which breeds in the wells. Failure to observe this results in a fine. Involving the persons using *An. gambiae* s.l. larval habitats in larval control efforts may lead to a more effective programme for the control of mosquitoes and hence of malaria.

## Conclusions

In conclusion, it is evident from the present study that larval abundance, survival and production of adult mosquitoes in irrigated vegetable farms such as the one in Gyinyase which is typical of most of such farms in the city of Kumasi and perhaps elsewhere, are quite high and, therefore, contribute significantly to adult mosquito populations and hence malaria transmission in the city. This supports an earlier observation by Afrane *et al.,*[4] that irrigation schemes such as those created for vegetable farming produce over 80% of malaria vectors which are involved in the transmission of malaria in the city of Kumasi.

## Competing interests

We declare that we have no conflicts or competing interest. All authors declare that we all had full access to all of the data in the study and take responsibility for the integrity of the data and the accuracy of the data analysis.

## Authors’ contributions

YAA participated in the study design and was responsible for data collection, management, and analysis, and drafting and producing the final manuscript; BWL and TK participated in the study design, supervised data collection and participated in producing final manuscript, RB participated in the study design and was part of data collection and management, and drafting and producing the final manuscript. GY designed the study, supervised data collection, and helped in drafting and producing the final manuscript. All authors read and approved the final version of the manuscript.

## Funding

This study was supported by the National Institute of Health (R01 AI094580, D43 TW01505 and R01 A150243).
